# Genome-Wide Identification of *YABBY* Genes in Orchidaceae and Their Expression Patterns in *Phalaenopsis* Orchid

**DOI:** 10.3390/genes11090955

**Published:** 2020-08-19

**Authors:** You-Yi Chen, Yu-Yun Hsiao, Song-Bin Chang, Diyang Zhang, Si-Ren Lan, Zhong-Jian Liu, Wen-Chieh Tsai

**Affiliations:** 1 Department of Life Sciences, National Cheng Kung University, Tainan 701, Taiwan; ricky80304@gmail.com (Y.-Y.C.); sbchang@mail.ncku.edu.tw (S.-B.C.); 2 Orchid Research and Development Center, National Cheng Kung University, Tainan 701, Taiwan; yunhsiao@gmail.com; 3 Institute of Tropical Plant Sciences and Microbiology, National Cheng Kung University, Tainan 701, Taiwan; 4 Key Lab of National Forestry and Grassland Administration for Orchid Conservation and Utilization at College of Landscape Architecture, Fujian Agriculture and Forestry University, Fuzhou 350002, China; deanyful@gmail.com (D.Z.); lkxz@fafu.edu.cn (S.-R.L.); 5 Zhejiang Institute of Subtropical Crops, Zhejiang Academy of Agricultural Sciences, Wenzhou 325005, China; 6 Center for Biotechnology and Biomedicine, Shenzhen Key Laboratory of Gene and Antibody Therapy State Key laboratory of Health Sciences and Technology (Prep), Tsinghua Shenzhen International Graduate School, Tsinghua University, Shenzhen 518055, China; 7 Henry Fok College of Biology and Agriculture, Shaoguan University, Shaoguan 512005, China

**Keywords:** Orchidaceae, *Phalaenopsis equestris*, *YABBY* gene, genome-wide, expression pattern

## Abstract

The plant *YABBY* transcription factors are key regulators in the lamina development of lateral organs. Orchid is one of the largest families in angiosperm and known for their unique floral morphology, reproductive biology, and diversified lifestyles. However, nothing is known about the role of *YABBY* genes in orchids, although biologists have never lost their fascination with orchids. In this study, a total of 54 *YABBY* genes, including 15 genes in CRC/DL, eight in INO, 17 in YAB2, and 14 in FIL clade, were identified from the eight orchid species. A sequence analysis showed that all protein sequences encoded by these YABBY genes share the highly conserved C2C2 zinc-finger domain and YABBY domain (a helix-loop-helix motif). A gene structure analysis showed that the number of exons is highly conserved in the same clades. The genes in YAB2 clade have six exons, and genes in CRC/DL, INO, and FIL have six or seven exons. A phylogenetic analysis showed all 54 orchid *YABBY* genes could be classified into four major clades, including CRC/DL, INO, FIL, and YAB2. Many of orchid species maintain more than one member in CRC/DL, FIL, and YAB2 clades, implying functional differentiation among these genes, which is supported by sequence diversification and differential expression. An expression analysis of *Phalaenopsis* *YABBY* genes revealed that members in the CRC/DL clade have concentrated expressions in the early floral development stage and gynostemium, the fused male and female reproductive organs. The expression of *PeINO* is consistent with the biological role it played in ovule integument morphogenesis. Transcripts of members in the FIL clade could be obviously detected at the early developmental stage of the flowers. The expression of three genes, *PeYAB2,* *PeYAB3*, and *PeYAB4*, in the YAB2 clade could be revealed both in vegetative and reproductive tissues, and *PeYAB4* was transcribed at a relatively higher level than that of *PeYAB2* and *PeYAB3*. Together, this comprehensive analysis provides the basic information for understanding the function of the *YABBY* gene in Orchidaceae.

## 1. Introduction

The small plant-specific *YABBY* gene family, belonging in the subfamily of the zinc-finger superfamily, plays important roles in the development of lateral organs [1], establishment of adaxial–abaxial polarity [2], leaf margin establishment [3], and stress response [4]. The members of which encode a class of transcription factors containing two conserved domains, which are a N-terminal Cys2 Cys2 zinc-finger motif and C-terminal helix-loop-helix YABBY domain [5,6]. Five subfamilies, including CRABS CLAW (CRC), FILAMENTOUS FLOWER (FIL)/YABBY3 (YAB3), INNER NO OUTER (INO), YABBY2 (YAB2), and YABBY5 (YAB5), are classified among extant angiosperms [7,8]. The genome of *Arabidopsis thaliana* encodes for six YABBY members (FIL, CRC, INO, YAB2, YAB3, and YAB5), and *Orzya sativa* has eight. It has been indicated that, before the diversification of the angiosperms, four gene duplication events have occurred in the *YABBY* gene family [9], leading to genes with both innovated and redundant functions.

Among the members of *YABBY* gene family, functional and expression characterizations suggest that CRC plays as a carpel development regulator across angiosperms [10,11] and nectaries in the core eudicots [12]. *Arabidopsis INO* is expressed limitedly in the abaxial domain of the outer integument and is important for the regulation of the outer integument development [13]. Conservation of the abaxial expression of *INO* orthologs in eudicots [14], eumagnoliids [15], and several basal angiosperm plants [16,17] has also documented. Members in the CRC and INO subfamilies have specified roles in reproductive organ development. Vegetative *YABBY* genes show functional redundancy during leaf development in *Arabidopsis* [18], as well as in other core eudicots [5] and monocots [19]. However, various expression patterns could also be observed in vegetative YABBY genes among monocot species. For example, maize *ZYB9* and *ZYB14* (*FIL/YAB3*-like genes) are expressed adaxially and may act a regulator in lateral outgrowth [19]. *OsYABBY1*, a *YAB2*-like gene from rice, is expressed in precursor cells that give rise to abaxial sclerenchyma in the leaves, the mestome sheath in the large vascular bundle, and sclerenchymatous cells in the palea and lemma of the flower and is thus suggested to determine the differentiation of certain cell types [20].

Containing more than 900 genera and 27,000 species [21], the Orchidaceae represents about 10% of the angiosperm plants and the largest family in species number. The Orchidaceae comprise five subfamilies, including Apostasioideae, Cypripedioideae, Vanilloideae, Orchidoideae, and Epidendroideae. They show a wide diversity of 70% epiphytic, 25% terrestrial, and 5% on various supports of vegetative growth and have successfully colonized almost every habitat on Earth [22]. Especially, they are known for their unique floral morphology and reproductive biology. Their flowers possess several reliable floral morphological synapomorphies, including a gynostemium, fused by the style and at least part of the androecium, and a highly evolved petal, the labellum [23]. In addition, many of the orchids show their mature pollen grains packaged as pollinia, and their ovary/ovule development is precisely triggered by the deposition of pollinia into the stigmatic cavity of gynostemium [24]. Evidence from flowering plants has shown that the *YABBY* gene family plays important roles in vegetative and reproductive developments. However, nothing is known about the role of *YABBY* genes in orchids, although biologists have never lost their fascination with orchids. Recently, several whole genomes of orchid species, including *Apostasia shenzhnica* (Apostasioideae) [25], *Phalaenopsis equestris* (Epidendroideae) [26], *Dendrobium catenatum* (Epidendroideae) [27], and *Gastrodia elata* (Epidendroideae) [28], have been sequenced. The completed assembly of the whole genome of these orchid species provides an opportunity for the systematic study of the orchid *YABBY* family. The aim of this study was to identify and compare *YABBY* genes at a genome-wide scale in orchids. In addition, the expression patterns of eight putative *Phalaenopsis YABBY* genes were analyzed using qRT-PCR in various tissues/organs.

## 2. Materials and Methods

### 2.1. Plant Materials

The species *Phalaenopsis aphrodite* subsp. *formosana* were collected from Chain-Port Orchids Nursery (Pingtung, Taiwan). All *Phalaenopsis* plants were grown in a glasshouse at National Cheng Kung University (NCKU) under natural light (photosynthetic photon flux density, 90 μmol m^−2^ s^−1^) and controlled temperature from 25 °C to 30 °C.

### 2.2. Sample Collection and RNA Preparation

**Figure 1:**
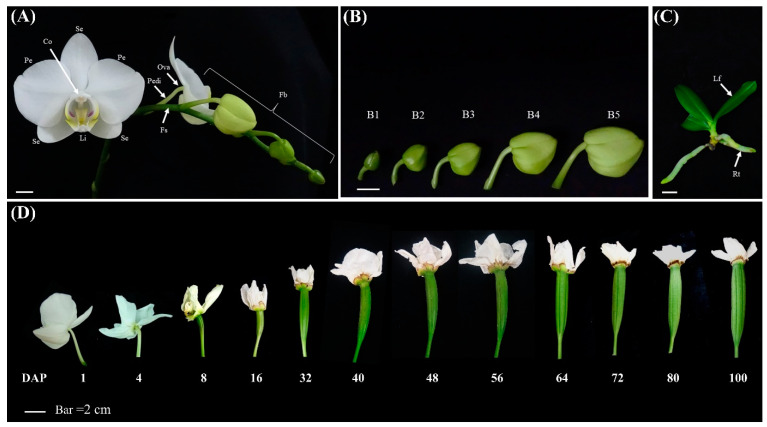
Morphology of *Phalaenopsis aphrodite* subsp. *formosana.* (**A**) Fully blooming flowers with floral buds and floral stalks in *Phalaenopsis* orchids. Scale bar = 1 cm. (**B**) Flower buds. Scale bar = 1 cm. (**C**) Leaf and root. Scale bar = 1 cm. (**D**) Developing ovary. Scale bar = 2 cm. Se, sepals; Pe, petals; Li, lip; Co, column (gynostemium); Pedi, pedicel; Fs, floral stalk; B1–B5, stage 1 to stage 5 floral buds; Lf, leaf; Rt, root; Ova, ovary; and DAP, days after pollination.

The flower buds were defined as B1 (0.5–1.0 cm), B2 (1.0–1.5 cm), B3 (1.5–2.0 cm), B4 (2.0–2.5 cm), and B5 (2.5–3.0 cm) stages (Figure 1A,B), accordingly [29]. The vegetative tissue (pedicel, floral stalk, leaf seedlings, and root seedlings) and floral organs (sepal, petal, labellum, and gynostemium) were collected (Figure 1A,C). Developing ovary and ovule from 0 to 100 days after pollination (DAP) were collected, as described by Chen et al. (Figure 1D) [30]. For total RNA preparation, all of *Phalaenopsis* orchid samples were quickly frozen in liquid nitrogen before extraction and stored at −80 °C for further use. Total RNA was extracted following the guanidium thiocyanate method described by O’Neill et al. [31].

### 2.3. Identification of YABBY Genes in Orchids

To identify *YABBY* genes in orchids, the Hidden Markov Model (HMM) profile of the Pfam YABBY domain (PF04690) was performed, respectively, against predicted proteomes of *P. equestris*, *D. catenatum*, *G. elata*, and *A. shenzhenica* [25,26,27,28]. For a more comprehensive collection of *YABBY* genes from other orchid species, several ongoing whole-genome sequencing data of orchid species, including *Vanilla shenzhenica* (Vanilloideae), *V. pompona*, *Platanthera zijinensis* (Orchidoideae), and *P. guangdongensis* were further searched. All identified orchid YABBY sequences were further confirmed by the blastp program in the NCBI database (https://www.ncbi.nlm.nih.gov/) for checking both zinc-finger and YABBY domains existing in each sequence.

### 2.4. Sequence Alignment

Multiple sequences alignment of full-length YABBY protein sequences of the orchid and other plants was calculated by the Align X program provided in the software Vector NTI package (Invitrogen, Carlsbad, CA, USA, Version 10). To investigate the conservation of the C2C2 zinc-finger domain and YABBY domain in orchid YABBY proteins and rice, the online tool WEBLOGO (https://weblogo.berkeley.edu/logo.cgi) [32] was adopted, and the multiple sequence alignment results were used as the input file. 

### 2.5. Gene Structure Analysis

The exon positions and sizes of identified orchid *YABBY* genes were acquired from the gff3 files (https://m.ensembl.org/info/website/upload/gff3.html). The identified *YABBY* genes were further compared to the assembled transcriptomic sequences. If the identified *YABBY* gene-coding sequence was inconsistent with the correspondent mRNA sequence, the correspondent mRNA sequence was mapped to the assembled genomic sequence, and the gene structure was edited manually. All of the exon numbers and lengths are listed in Table 1.

### 2.6. Phylogenetic Analysis

Before the phylogenetic analysis, a multiple sequence alignment of full-length YABBY proteins was performed by using ClustalW (Hinxton, Cambridgeshire, UK) with the default parameters. The phylogenetic trees were built using the MEGA6 Neighbor-Joining method with the Dayhoff model and pairwise deletion option parameters [33]. A bootstrap analysis was performed using 1000 iterations. The eudicots and monocots of YABBY proteins were collected from the NCBI database. All of the protein accession numbers are listed in Supplementary Table S1.

### 2.7. Real-Time RT-PCR

After RNA extraction, total RNA was treated with RNase-free DNase (NEB, Hertfordshire, UK) following the manufacturer’s protocols to remove contaminating genomic DNA. Reverse transcription to cDNA was synthesized using the Superscript II kit (Invitrogen, Carlsbad, CA, USA). Real-time RT-PCR was performed on the ABI 7500, Applied Biosystems System using SYBR GREEN PCR Master Mix (Applied Biosystems, Warrington, UK). PCR was performed with the following reaction conditions: 95 °C for 10 min, 40 cycles of 95 °C for 15 s, and 60 °C for 1 min. All the raw data were analyzed with the Sequencing Detection System v1.2.3 (Applied Biosystems). *PeActin4* (PACT4, AY134752) was used for normalization [34]. Mean and standard error were calculated from three biological and technical replicates. Primers used for real-time RT-PCR were designed by using Primer Express 3.0 (Thermo Fisher Scientific, Foster City, CA, USA) and listed in Supplementary Table S2.

## 3. Results and Discussion

### 3.1. Identification and Sequence Analyses of YABBY Genes from Orchidaceae

To identify the *YABBY* genes from orchids, the YABBY domain conserved sequence (PF04690) generated from the Pfam protein family database was used to search the orchid predicted proteomes. Eight members of YABBY family were identified in the genome of *P. equestris*, eight in *D. catenatum*, six in *A. shenzenica*, and five in *G. elata*. For a more comprehensive collection of *YABBY* genes from other orchid species, several ongoing whole-genome sequencing data of orchid species, including *V. shenzhenica*, *V. pompona*, *P. zijinensis*, and *P. guangdongensis*, were further searched. In two *Vanilla* species, seven genes of *YABBY* family were identified in their respective genomes. In addition, seven and six *YABBY* genes could be identified from *P. zijinensis* and *P. guangdongensis*, respectively. In total, 54 predicted *YABBY* genes were obtained from Orchidaceae. 

**Figure 2:**
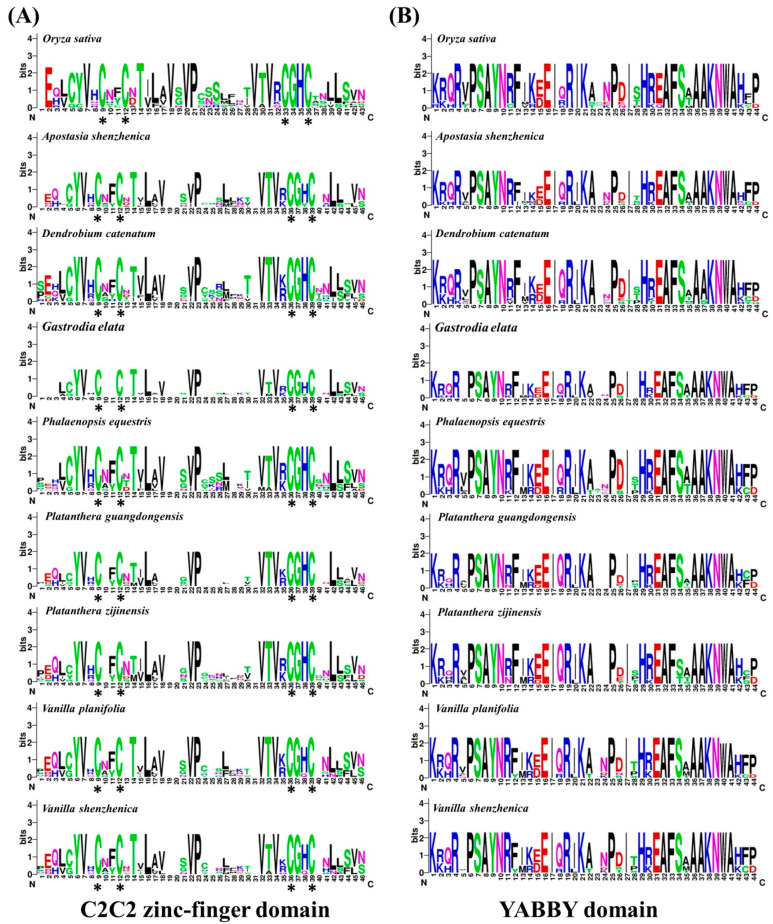
Conserved domains of orchid and rice YABBY protein sequences. (**A**) Sequence logo revealing the N-terminal conserved zinc-finger domain. Conserved cysteine residues in the zinc-finger domain are indicated with black asterisks. (**B**) Sequence logo showing the C-terminal conserved YABBY domain.

Multiple sequence alignments were performed to generate sequence logos of both C2C2 and YABBY domains in eight orchid species and rice (Figure 2). The results showed that both the C2C2 zinc-finger and YABBY domain are highly conserved in rice and orchid plants (Figure 2). Additionally, the YABBY domain is more conserved than the C2C2 zinc-finger domain (Figure 2). We also compared the amino acid sequences encoded in orchid *YABBY* genes with two Arabidopsis YABBY proteins (AtCRC and AtINO) (Figure S1). As shown in Figure S1, we found not only cysteine residues at the expected positions but, also, that valine, leucine, proline, valine, and glycine (indicated by the triangle) at the zinc-finger domain are completely conserved among *Arabidopsis* and eight orchid species. In addition, the distributions of the conserved YABBY domain with a helix-loop-helix motif were significantly conserved in rice and eight orchid species (Figure 2 and Figure S2–S5). 

### 3.2. Phylogenetic Analysis of YABBY Genes Orchids among Orchid and Other Angiosperm Plants

**Figure 3:**
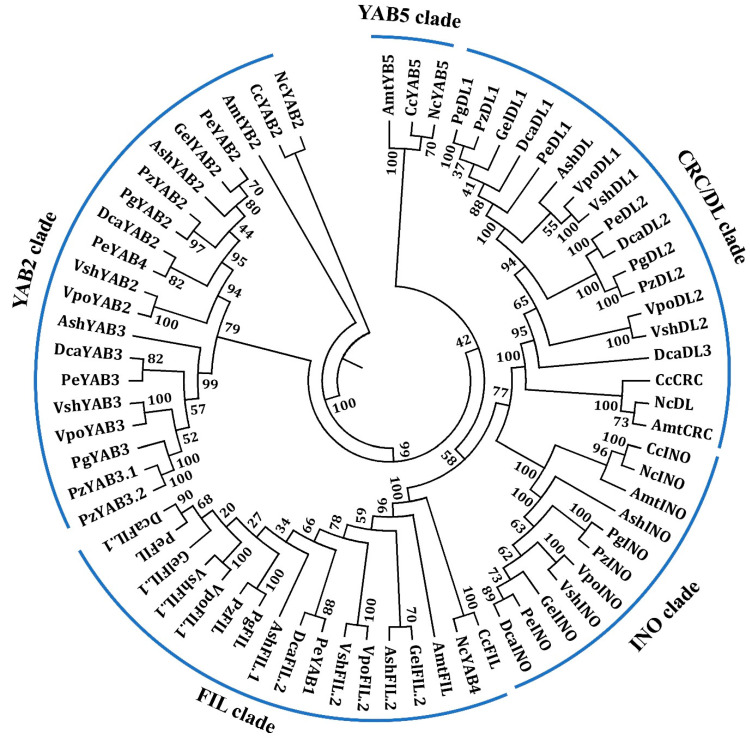
Phylogenetic tree of YABBY proteins from *Amborella trichopoda*, *Cabomba caroliniana*, *Nymphaea colorata*, and different types of orchids. The phylogenetic tree was constructed with the neighbor-joining (NJ) method in MEGA 6.0 software and was divided into five subgroups. A bootstrap analysis was conducted with 1000 replications. Gel, *Gastrodia elata*; Vpo, *Vanilla planifolia*; Pe, *Phalaenopsis equestris*; Vsh, *Vanilla shenzhenica*; Pg, *Platanthera guangdongensis*; Pz, *Platanthera zijinensis*; Dca, *Dendrobium catenatum*; Ash, *Apostasia shenzhenica*; Amt, *Amborella trichopoda*; Cc, *Cabomba caroliniana*; and Nc, *Nymphaea colorata*. The protein accession numbers for the related proteins are listed in Supplementary Table S1.

To analyze the evolutionary relationships among these orchid *YABBY* genes, we constructed a phylogenetic tree based on the full-length sequences of YABBY proteins together with YABBY proteins from *Amborella trichopoda*, *Cabomba caroliniana*, and *Nymphaea colorata*, the first diverging angiosperm lineages [17,35,36]. The phylogenetic tree indicated that the orchid YABBY proteins could be divided into four clades, named the CRC/DL, INO, FIL, and YAB2, with strong bootstrap value support (Figure 3). We did not find orchid gene-encoded proteins belonging to YAB5 clade (Figure 3). This result suggested that YAB5-related genes might be lost in Orchidaceae. Fifteen of orchid YABBY proteins (GelDL1, VpoDL2, VpoDL1, PeDL2, PeDL1, VshDL2, VshDL1, PgDL2, PgDL1, PzDL2, PzDL1, DcaDL3, DcaDL2, DcaDL1, and AshDL) were in the CRC/DL clade; eight proteins (GelINO, VpoINO, PeINO, VshINO, PgINO, PzINO, DcaINO, and AshINO) in the INO clade; fourteen (GelFIL.1, GelFIL.2, VpoFIL.1, VpoFIL.2, PeYAB1, PeFIL, VshFIL.1, VshFIL.2, PgFIL, PzFIL, DcaFIL.1, DcaFIL.2, AshFIL.1, and AshFIL.2) in the FIL clade; and seventeen (AshYAB2, AshYAB3, DcaYAB2, DcaYAB3, GelYAB2, PeYAB2, PeYAB3, PeYAB4, PgYAB2, PgYAB3, PzYAB2, PzYAM3.1, PzYAM3.2, VpoYAB2, VpoYAB3, VshYAB2, and VshYAB3) in the YAB2 clade (Figure 3).

**Figure 4:**
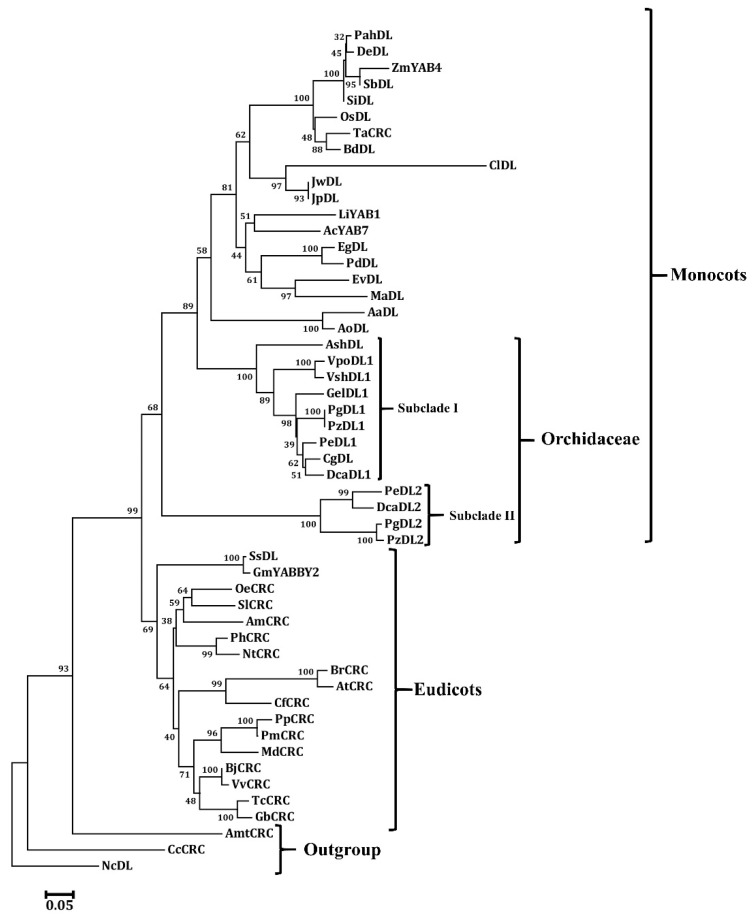
Phylogenetic tree of the CRC/DL protein from the angiosperms. The phylogenetic tree was constructed with the neighbor-joining (NJ) method in MEGA 6.0 software (Phoenix, AZ, USA). A bootstrap analysis was conducted with 1000 replications. The protein accession numbers for the related proteins are listed in Supplementary Table S1.

To further investigate the evolutionary relationships among orchid YABBY proteins, the YABBY protein sequences of eudicots and monocots were retrieved from the public database for further analysis. We found that each orchid species contains two members of the CRC/DL clade, except that *Apostasia* and *Gastrodia*, respectively, has only one and *Dendrobium* has three members (Table 1). Owing to that *VpoDL2* and *VshDL2* from *Vanilla* and *DcaDL3* from *Dendrobium* have variable exon numbers and/or exon lengths (Table 1), these genes were excluded for further phylogenetic analyses. The phylogenetic analysis of CRC/DL showed that orchid CRC/DL could be divided into two subclades (Figure 4). Orchid subclade I is grouped with monocot CRC/DL members supported by high bootstrap values, and orchid subgroup II is positioned at the base of the monocots with moderate bootstrap values (Figure 4). This result suggests that a duplication event might occur at the most recent common ancestor of the monocots. Orchid CRC/DL-like proteins could be divided into subclade I and subclade II (Figure 4). In addition, most monocot and orchid plants, *Apostasia* and *Gastrodin*, might lose their members in subclade II (Figure 4). We also re-added VpoDL2 and VshDL2 for the phylogenetic analysis. The tree topology is similar to that shown in Figure 4, though the support of VpoDL2 and VshDL2 located in subclade II was not significant (Figure S6). 

**Figure 5:**
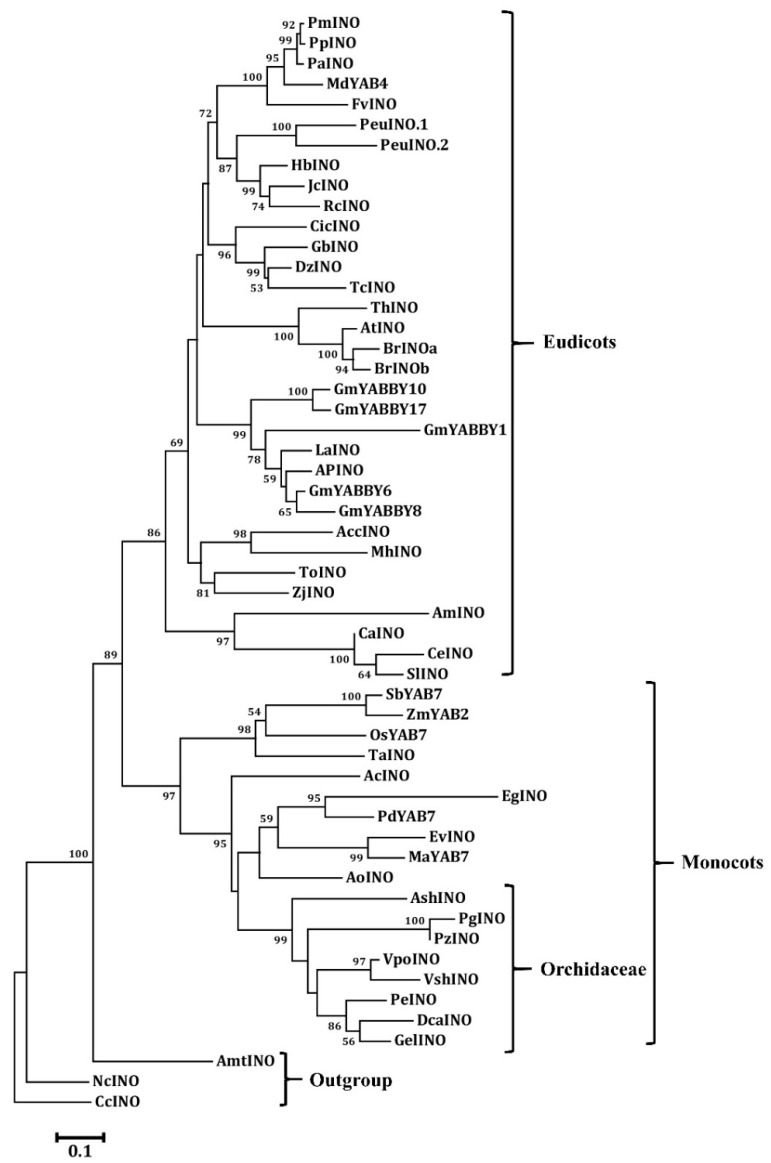
Phylogenetic tree of the INO protein from the angiosperms. The phylogenetic tree was constructed with the neighbor-joining (NJ) method in MEGA 6.0 software. A bootstrap analysis was conducted with 1000 replications. The protein accession numbers for the related proteins are listed in Supplementary Table S1.

We found each orchid species contains one member of the INO clade as the other monocot and eudicot species has. All of the orchid INO members were grouped together, with very high bootstrap value support (Figure 5). It is possible that the group has not undergone expansion, and group members may perform biologically conserved functions. 

**Figure 6:**
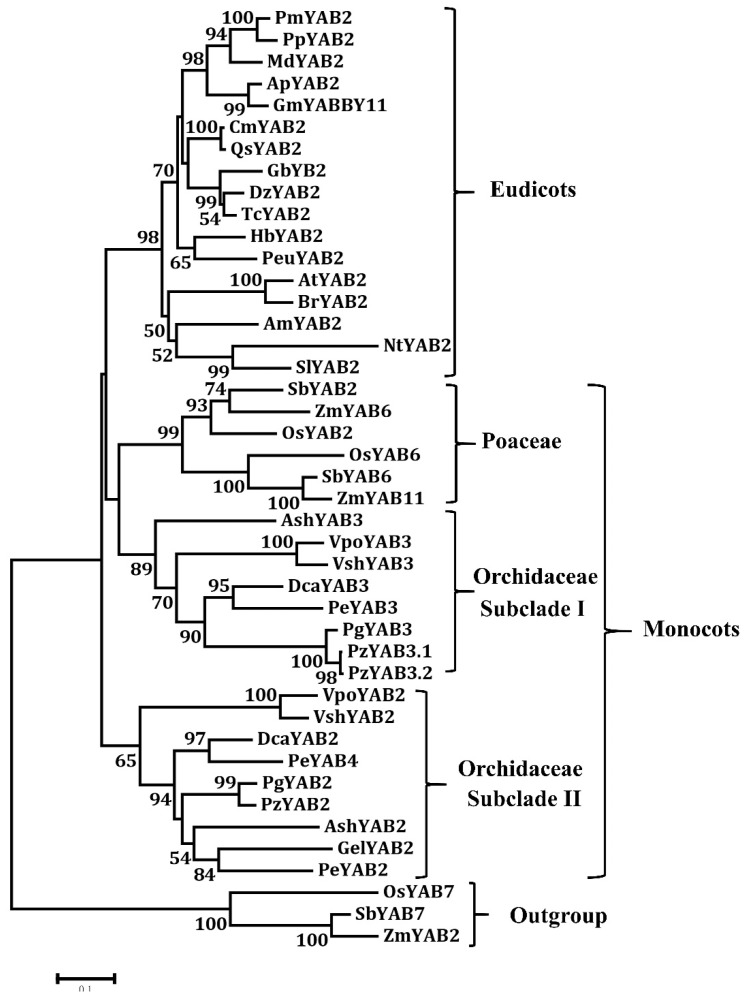
Phylogenetic tree of the YAB2 protein from the angiosperms. The phylogenetic tree was constructed with the neighbor-joining (NJ) method in MEGA 6.0 software. A bootstrap analysis was conducted with 1000 replications. The protein accession numbers for the related proteins are listed in Supplementary Table S1.

The orchid genes in the YAB2 clade can be divided into two subclades (Figure 6). Members in the orchid subclade I are sister to the YAB2 members of Poaceae. Members in the Orchid subclade I and Poaceae are sister to those of eudicot plants (Figure 6). Relationships among the orchid subclade I, Poaceae, and eudicots are very robust in this analysis. These results suggest that these genes have similar functions involved in leaf development. Noticeably, neither eudicot nor monocot YAB2 members are grouped with members in the orchid subclade II (Figure 6), suggesting that YAB2 in orchid subclade II has particular functions in orchid plants. Interestingly, *G. elata*, a fully mycoheterotrophic plant without leaves [28], does not have members in subclade I (Figure 6). It is reasonable that *G. elata* lost YAB2 in subclade I, causing the defect of leaf development. 

**Figure 7:**
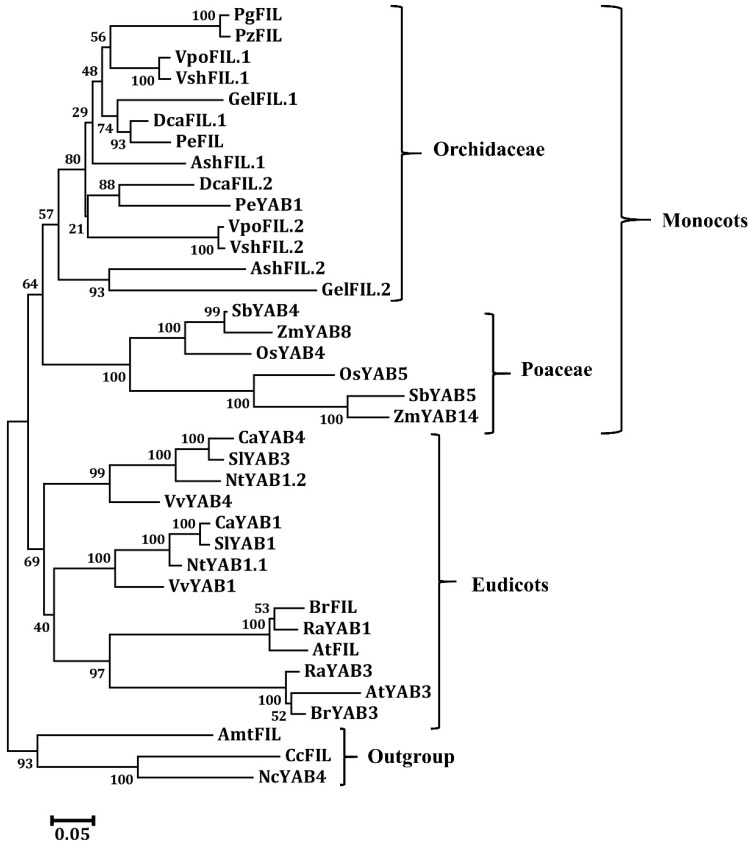
Phylogenetic tree of the FIL protein from the angiosperms. The phylogenetic tree was constructed with the neighbor-joining (NJ) method in MEGA 6.0 software. A bootstrap analysis was conducted with 1000 replications. The protein accession numbers for the related proteins are listed in Supplementary Table S1.

It is noteworthy that *FIL*-like genes have undergone expansion in eudicots and monocots, and we found that each orchid species has two copies of *FIL*-like genes, except for the two species in *Platanthera* (Orchidoideae). The most parsimonious interpretation of this observation is that the two *FIL*-like genes were generated by the whole-genome duplication event that occurred at the most recent common ancestor of orchids [25], though the phylogenetic tree was not obviously indicated (Figure 7). The *Platanthera* species contain only one copy of *FIL*-like genes, suggesting that the other gene has been lost in the *Platanthera* genus or in the most recent common ancestor of Orchidoideae.

### 3.3. Gene Structure Analysis of YABBY Genes in Orchids

To further investigate the phylogenetic relationship, we compared YABBY gene structures in eight orchid species. Previous reports described that the rice and *Cabomba* YABBY genes showed highly similar exon patterns [17,20]. In this study, we found that the orchids *YABBY* genes in the CRC/DL, INO, YAB2, and FIL clades have six to seven exons (Table 1). The fourth and fifth exons constituting YABBY domain have the exact same lengths (exon 4 and 49 bp and exon 5 and 76 bp, Table 1). In general, genes close to each other in the phylogenetic tree showed highly similar exon numbers and lengths. For example, most of genes in the CRC/DL, FIL, and INO clades have seven exons, and all genes in the YAB2 clade have six exons. The first exon length of most genes in the CRC/DL clade is 69 bp and is larger than 100 bp in the FIL clade members (Table 1). Many genes in the CRC/DL clade have six exon lengths 87 or 84 bp, and those in the FIL clade have 75 bp in six exons (Table 1). 

### 3.4. Gene Expression Analysis in Phalaenopsis Orchid

**Figure 8:**
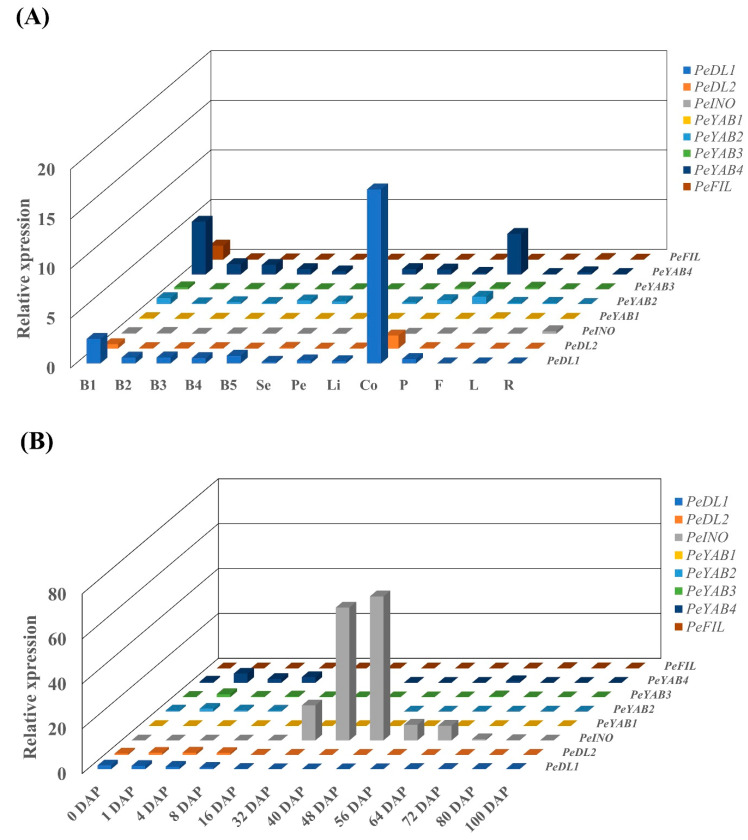
Expression patterns of *PeYABBY*-related genes in *P. aphrodite* subsp. *formosana*. (**A**) Expression patterns of *PeYABBY*-related genes in various organs. (**B**) Expression patterns of *PeYABBY*-related genes in various ovule developmental stages. B1–B5, stage 1 to stage 5 floral buds; Se, sepals; Pe, petals; Li, lip; Co, column (gynostemium); P, pedicel; F, floral stalk; L, leaf; R, root; and DAP, days after pollination.

To explore and characterize the *YABBY* genes expression profiles in orchids, real-time RT-PCR was performed with use of the *Phalaenopsis* orchid as the model plant. The results showed that high expressions of *PeDL1*, *PeDL2*, and *PeINO* were detected at reproductive tissues. Both *PeDL1* and *PeDL2* transcripts could be significantly measured at stage 1 developing floral buds and gynostemium, and the expression of *PeDL1* is much higher than that of *PeDL2* in these two tissues/organs (Figure 8A and Figure S7). In addition, both of them could be expressed at early developmental stages of the ovule (0 to 8 days after pollination, DAP) (Figure 8B and Figure S8). Noticeably, a high expression of *PeINO* was specifically concentrated during 32 to 64 DAP of ovule development (Figure 8B and Figure S8). *Phalaenopsis* contains two genes, *PeFIL* and *PeYAB1*, in the FIL clade. Transcripts of *PeFIL* could be obviously detected at stage 1 developing floral buds. In general, *PeFIL* has higher expression levels than that of *PeYAB1* in any organs/tissues. The expression of three genes, *PeYAB2*, *PeYAB3*, and *PeYAB4*, in the YAB2 clade revealed variable profiles. *PeYAB4* was transcribed at a relatively higher level than that of *PeYAB2* and *PeYAB3*, though all of them shared overlapping expressions at tissues/organs (Figure 8, Figures S7 and S8). *PeYAB4* was majorly expressed at stage 1 developing floral buds, pedicel, and during 1 to 8 DAP of the developing ovule (Figure 8, Figures S7 and S8). These results suggest members in the YAB2 clade may have functions in both vegetative and reproductive tissues, and *PeYAB4* might play a more important role than that of *PeYAB2* and *PeYAB3*. To further define the expressions in Orchidaceae, the expression data for various tissues of *Dendrobium* and *Apostasia*, the FPKM values (flower bud, pollinium, gynostemium, seed, stem, and leaf) were downloaded from OrchidBase 4.0. (http://orchidbase.itps.ncku.edu.tw/est/home2012.aspx). The expression of orchid YABBY genes in reproductive tissues (flower bud, pollinium, and gynostemium) was higher than in vegetative tissues (stem, leaf, and root) (Figure S9). *DcaYAB2*, *DcaYAB3*, *DcaDL1*, *DcaDL2*, and *DcaDL3* also showed higher expressions in gynostemium (Figure S9). A similar expression pattern was observed in PeYAB2, *PeYAB3*, *PeDL1*, and *PeDL2* (Figure S9). A higher expression of orchid *YABBY* genes was observed in flower buds, with an exception of *AshINO* and *DcaINO*, which showed lower expressions in all the tissues (Figure S9).

## 4. Discussion

The YABBY transcription factors represent a small family in plants, whose members are characterized by two conserved domains: the C2C2 zinc-finger and YABBY with helix–loop–helix motif [37,38,39]. Five *YABBY* genes have been described in species *A. trichopoda* and *N. colorata* as representatives of two of the three orders of basal angiosperms (Amborellales and Nymphaeales, respectively), as well as in the species *C. caroliniana*, as a representative of the core eudicots. [17,35,36]. In the model eudicot plant *Arabidopsis*, six YABBY genes have been identified [13,37,40], and eight in the monocot representative—rice *Oryza sativa* [20]. The *YABBY* genes play important roles in regulating the development of lateral organs such as flowers, ovule, and leaves. In this study, five to eight *YABBY* genes were identified from each genome of eight orchid species. The number of *YABBY* genes that exist in each orchid genome could be comparable to that identified in the monocot and dicot species; an exception is the lack of YAB5-like genes in orchids. 

The phylogenetic tree showed that the *YABBY* genes can be divided into five groups (CRC/DL, INO, FIL, YAB2, and YAB5), but orchid *YABBY* genes are only found in four clades and absent in the YAB5 clade, which exclusively comprises *YABBY* genes of basal angiosperms and eudicots. It has been reported that monocot species, including rice and pineapple, also do not contain *YAB5*-like genes [41]. De Almeida et al. [42] studied the comparative development of the androecial form across the Zingiberales and found one *YABBY2* gene, which was also less homologous to *YAB5*. Based on this, they suggested that the duplication resulting in the separate *YABBY2* and *YABBY5* gene lineages most likely occurred after the divergence of monocots and eudicots [42]. This result suggested that the monocot plants might lose the genes in the YAB5 clade. The phylogenetic analysis also revealed that two *CRC/DL* paralogous genes were retained in many of the orchid species, and similar expression patterns of *Phalaenopsis PeDL1* and *PeDL2* were discovered that both genes majorly expressed in gynostemium. A gynostemium is a compound structure formed by stamens and pistils fused by filaments and style. Orchids have one, two, or three fertile abaxial stamens, which are invariably fused to the style [43]. The adaxial side of gynostemium is specialized to form a stigmatic cavity for providing an elaborate sticky space for accepting pollinium. Notably, only one *DL*/*CRC*-like gene was found in the genome of primitive *A. shenzhenica*, which had a gynostemium without stigmatic cavity formations. The retention of duplicated *DL*/*CRC* genes in the sister lineage to the Apostasioideae suggested an important role for *DL*/*CRC* genes in orchid gynostemium innovation. 

Orchids are unusual among flowering plants in that, in many species, the ovule is not mature at the time of pollination. Pollination-regulated ovule initiation and development in *Phalaenopsis* has been characterized [24,30]. We found a high expression of *PeINO* is restricted during the process of ovule development at 32 to 64 DAP (Figure 8B). The period of *PeINO* transcribed nicely fits the process of ovule integument development [30], suggesting that *PeINO* plays a vital role involved in ovule integument morphogenesis. Consistent results were observed where the expression of *INO*-like genes is confined to the outer integument of the ovule in *Arabidopsis* and several basal angiosperms [13,16,17]. In this study, each of the orchid species examined only has one member in the INO clade. Together, the expression regulation and biological function of *INO*-like genes should be conserved in angiosperm plants.

*Arabidopsis FIL*, *YAB2*, and *YAB3* are expressed in cotyledons, leaves, and flowers and redundantly control these lateral organ developments [38,44,45]. The *FIL* orthologs of *TOB1*-related *YABBY* genes play similar functions in flower developments in rice [46]. Our results indicated that most of the orchid species retain two *FIL*-like and two or three *YAB2*-like genes. The *Phalaenopsis FIL*-like gene *PeFIL* conferred an 18-fold expression level higher than that of *PeYAB1* at the early developmental stage of floral buds (Figure S7), which suggests that the function of *PeFIL* and *PeYAB1* is redundant, and *PeFIL* might be more important than *PeYAB1* for floral development. The expression of *YAB2*-like genes could be detected both at reproductive and vegetative tissues, and *PeYAB4* expression showed higher than that of the other two genes (Figure 8). It is worth noticing that *G. elata* only has one *YAB2*-like gene, *GelYAB2*, classified into the orchid YAB2 subclade II (Figure 6). This orchid species has highly reduced leaves, and systematics often refer to the plants as leafless [28]. The evidence provided here implied that orchid *YAB2*-like genes in subclade I might be related to the determination of lamina outgrowth of the leaves. 

## 5. Conclusions

In this study, fifty-four YABBY genes were identified from eight orchid species and analyzed systematically in Orchidaceae. The orchid *YABBY* genes could be categorized into four major clades, including CRC/DL, INO, FIL, and YAB2. Many of the orchid species maintain more than one member in the CRC/DL, FIL, and YAB2 clades. Sequence and gene structure diversifications, as well as differential expressions of genes in these three clades, suggested functional differentiation among these genes. Our results represent a comprehensive genome-wide study of the YABBY gene family that opens the gate into further detailed studies on the gene function and evolution of YABBY genes in orchids.

## Figures and Tables

**Table 1:** The gene structures of the YABBY genes in the exon region.

Clades	Species	Gene Name	Exon 1	Exon 2	Exon 3	Exon 4	Exon 5	Exon 6	Exon 7
CRC/DL	*Apostasia shenzhenica*	*AshDL*	69 ^a^	129	94	49	76	93	93
	*Dendrobium catenatum*	*DcaDL1*	69	129	94	49	76	87	66
	*Gastrodia elata*	*GelDL1*	69	129	94	49	76	87	66
	*Phalaenopsis equestris*	*PeDL1*	69	129	94	49	76	87	66
	*Platanthera guangdongensis*	*PgDL1*	69	129	97	49	76	87	66
	*Platanthera zijinensis*	*PzDL1*	69	129	97	49	76	87	66
	*Vanilla planifolia*	*VpoDL1*	69	129	94	49	76	87	66
	*Vanilla shenzhenica*	*VshDL1*	69	129	94	49	76	87	66
	*Dendrobium catenatum*	*DcaDL2*	69	132	103	49	76	84	72
	*Dendrobium catenatum*	*DcaDL3*	69	120	82	49	76	102	66
	*Phalaenopsis equestris*	*PeDL2*	69	132	103	49	76	87	75
	*Platanthera guangdongensis*	*PgDL2*	72	141	106	49	76	84	69
	*Platanthera zijinensis*	*PzDL2*	72	141	106	49	76	84	69
	*Vanilla planifolia*	*VpoDL2*	69	114	82	49	76	60	
	*Vanilla shenzhenica*	*VshDL2*	69	114	82	49	76	198	
FIL	*Apostasia shenzhenica*	*AshFIL.1*	102	168	124	49	76	78	78
	*Apostasia shenzhenica*	*AshFIL.2*	108	138	118	49	76	129	
	*Dendrobium catenatum*	*DcaFIL.1*	105	159	130	49	76	75	75
	*Dendrobium catenatum*	*DcaFIL.2*	102	159	127	49	76	75	75
	*Gastrodia elata*	*GelFIL.1*	102	153	139	49	76	78	81
	*Gastrodia elata*	*GelFIL.2*	99	159	112	49	76	75	78
	*Phalaenopsis equestris*	*PeFIL*	105	159	127	49	76	75	78
	*Phalaenopsis equestris*	*PeYAB1*	102	159	127	49	76	75	75
	*Platanthera guangdongensis*	*PgFIL.1*	105	159	139	49	76	75	75
	*Platanthera zijinensis*	*PzFIL.1*	105	159	139	49	76	75	75
	*Vanilla planifolia*	*VpoFIL.1*	105	159	130	49	76	75	84
	*Vanilla planifolia*	*VpoFIL.2*	111	159	133	49	76	75	81
	*Vanilla shenzhenica*	*VshFIL.1*	105	159	130	49	76	75	84
	*Vanilla shenzhenica*	*VshFIL.2*	111	159	133	49	76	75	81
INO	*Apostasia shenzhenica*	*AshINO*	90	81	115	49	76	99	75
	*Dendrobium catenatum*	*DcaINO*	84	81	127	49	76	99	87
	*Gastrodia elata*	*GelINO*	87	81	136	49	76	105	
	*Phalaenopsis equestris*	*PeINO*	84	81	130	49	76	99	66
	*Platanthera guangdongensis*	*PgINO*	84	81	127	49	76	156	
	*Platanthera zijinensis*	*PzINO*	84	81	127	49	76	126	
	*Vanilla planifolia*	*VpoINO*	87	81	133	49	76	90	96
	*Vanilla shenzhenica*	*VshINO*	87	81	133	49	76	90	96
YAB2	*Apostasia shenzhenica*	*AshYAB2*	69	123	121	49	76	111	
	*Dendrobium catenatum*	*DcaYAB2*	69	120	106	49	76	114	
	*Gastrodia elata*	*GelYAB2*	66	123	103	49	76	117	
	*Phalaenopsis equestris*	*PeYAB2*	69	126	118	49	76	114	
	*Phalaenopsis equestris*	*PeYAB4*	69	120	118	49	76	69	
	*Platanthera guangdongensis*	*PgYAB2*	84	117	118	49	76	36	
	*Platanthera zijinensis*	*PzYAM2*	105	117	118	49	76	36	
	*Vanilla planifolia*	*VpoYAB2*	69	120	85	49	76	114	
	*Vanilla shenzhenica*	*VshYAB2*	69	120	85	49	76	114	
	*Apostasia shenzhenica*	*AshYAB3*	78	120	88	49	76	111	
	*Dendrobium catenatum*	*DcaYAB3*	78	120	124	49	76	48	
	*Phalaenopsis equestris*	*PeYAB3*	78	120	124	49	76	81	
	*Platanthera guangdongensis*	*PgYAB3*	78	123	133	49	76	108	
	*Platanthera zijinensis*	*PzYAM3.1*	78	123	133	49	76	108	
	*Platanthera zijinensis*	*PzYAM3.2*	78	123	133	49	76	108	
	*Vanilla planifolia*	*VpoYAB3*	78	120	127	49	76	117	
	*Vanilla shenzhenica*	*VshYAB3*	78	120	127	49	76	105	

^a^ The number represents the length of the exons in base pairs.

## Supplementary Materials

The following are available online at https://www.mdpi.com/2073-4425/11/9/955/s1, Supplemental Figure S1: Conserved C2C2 zinc-finger and YABBY domains of YABBY proteins among *A. thaliana* and eight orchid species. Supplemental Figure S2: Multiple sequence alignment of the CRC/DL-related protein among eight orchid species. Supplemental Figure S3: Multiple sequence alignment of the INO-related protein among eight orchid species. Supplemental Figure S4: Multiple sequence alignment of YAB2-related protein among eight orchid species. Supplemental Figure S5: Multiple sequence alignment of the FIL-related protein among eight orchid species. Supplemental Figure S6: Phylogenetic tree of the CRC/DL protein from the angiosperms. Supplemental Figure S7: Quantitative real-time PCR analysis of the expressions of *PeYABBY* genes in *P. aphrodite* subsp. *formosana* at various organs. Supplemental Figure S8: Quantitative real-time PCR analysis of the expressions of *PeYABBY* genes in *P. aphrodite* subsp. *formosana* at various ovule developmental stages. Figure S9: Expression patterns of *YABBY* genes in different tissues of *Apostasia*, *Dendrobium*, and *Phalaenopsis*. Supplemental Table S1: YABBY-related genes in the phylogenetic tree and protein alignments. Supplementary Table S2: List of primers used in this work.
