# Supplementary material for: Genome-Wide Identification of YABBY Genes in Orchidaceae and Their Expression Patterns in Phalaenopsis Orchid
Source: Genes (Basel). 2020 Aug 19;11(9):955. doi: 10.3390/genes11090955 (PMC7563141; doi:10.3390/genes11090955)
Supplement: Supplementary file 1 [file genes-11-00955-s001.zip › genes-872759-suppl/Supplementary_File_1_Supplemental_Fig_1-9.pdf]

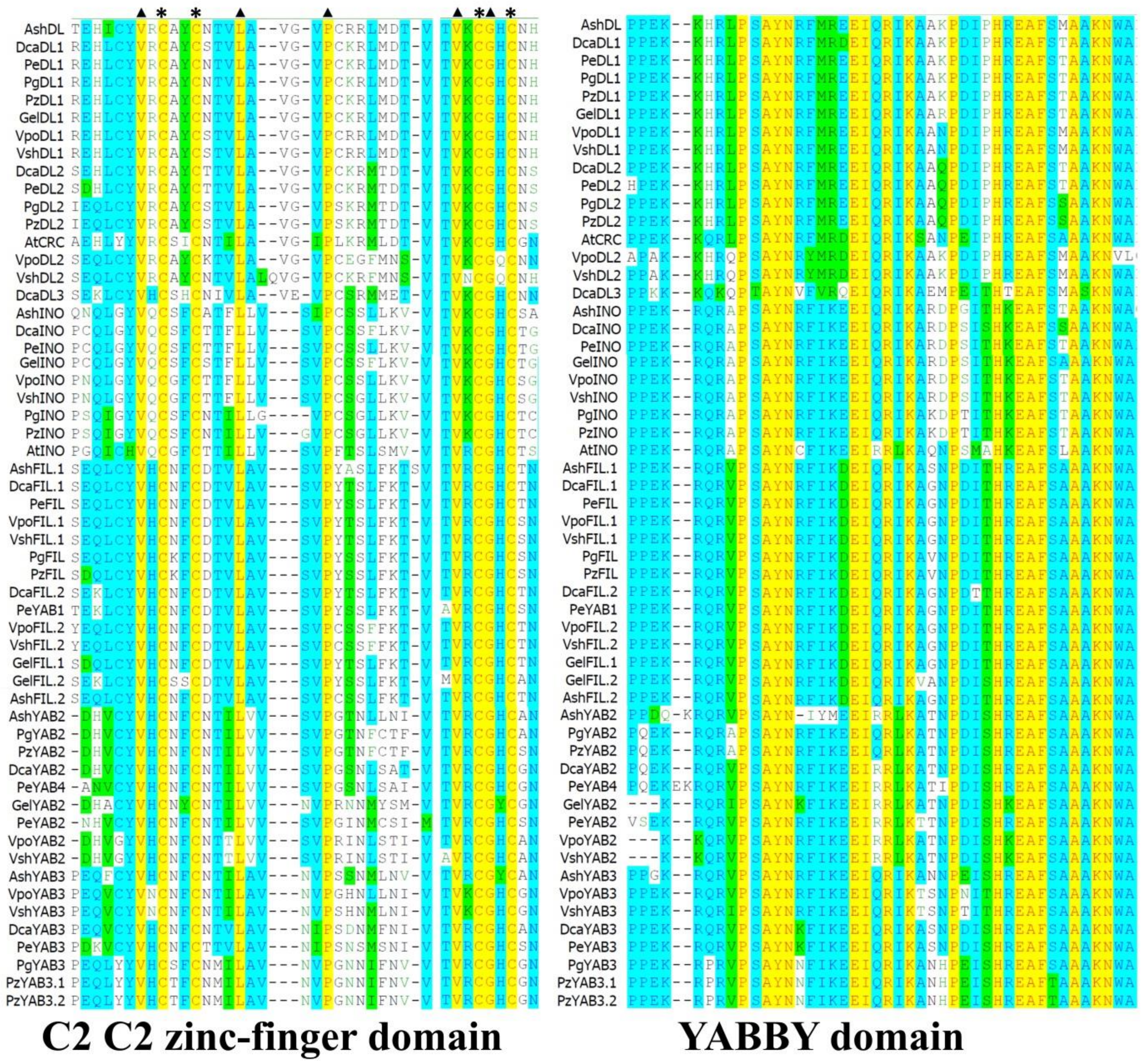

Supplemental Fig. 1. Conserved C2C2 zinc-finger and YABBY domains of YABBY proteins among *A. thaliana* and eight orchid species.

Conserved cysteine residues in the zinc-finger domain are marked by black asterisks. Black triangles indicate the other completely conserved residues existed in C2C2 zinc-finger domain. Gel, *G. elata*; Vpo, *V. planifolia*; Pe, *P. equestris;* Vsh, *V. shenzhenica*; Pg, *P. guangdongensis*; Pz, *P. zijinensis*; Dca, *D. catenatum*; Ash, *A. shenzhenica*; At, *A. thaliana*.

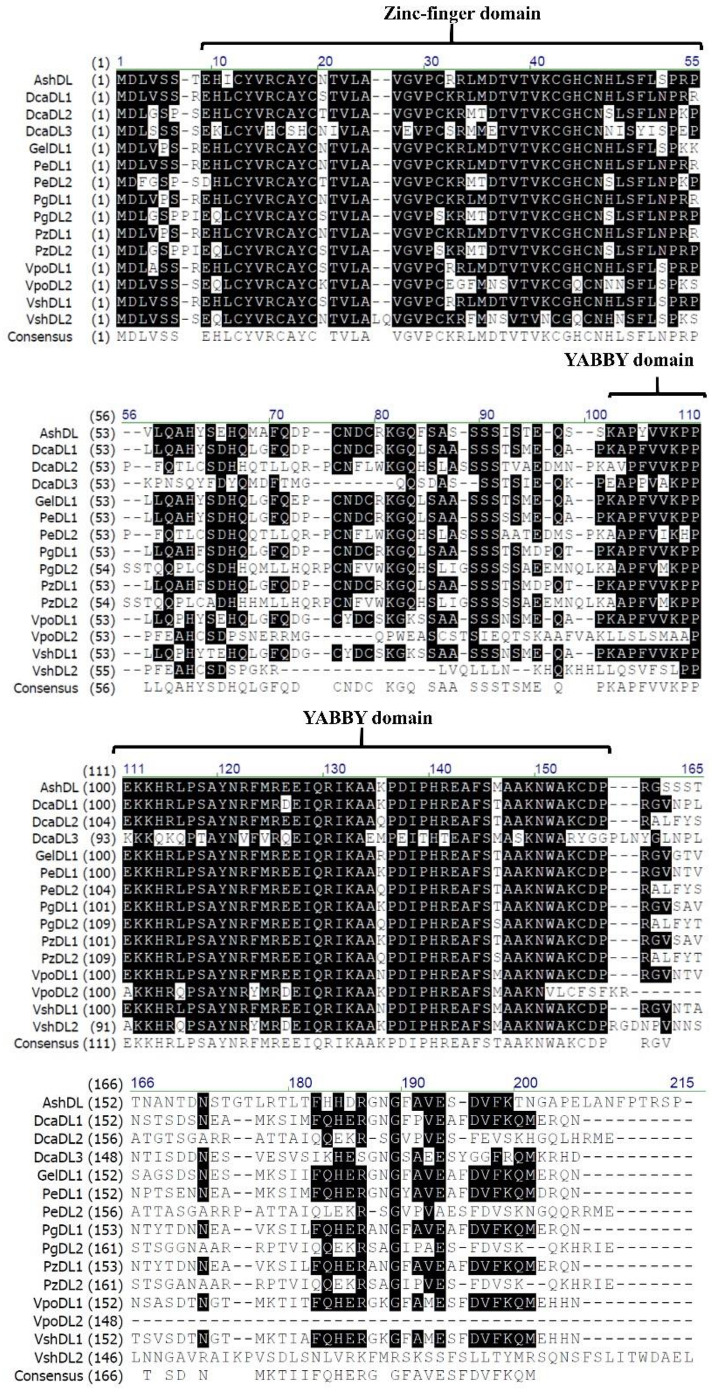

Supplemental Fig. 2. Multiple sequence alignment of CRC/DL related protein among eight orchid species.

Multiple sequence alignment of the amino acid sequences of CRC/DL proteins was performed by using ClustalW with default settings. The black shade indicates identical amino acids. Two highly conserved C2C2 zinc-finger domain and YABBY domain are marked with black brackets. Gel, *G. elata*; Vpo, *V. planifolia*; Pe, *P. equestris*; Vsh, *V. shenzhenica*; Pg, *P. guangdongensis*; Pz, *P. zijinensis*; Dca, *D. catenatum*; Ash, *A. shenzhenica*. Accession numbers are listed in Table S1.

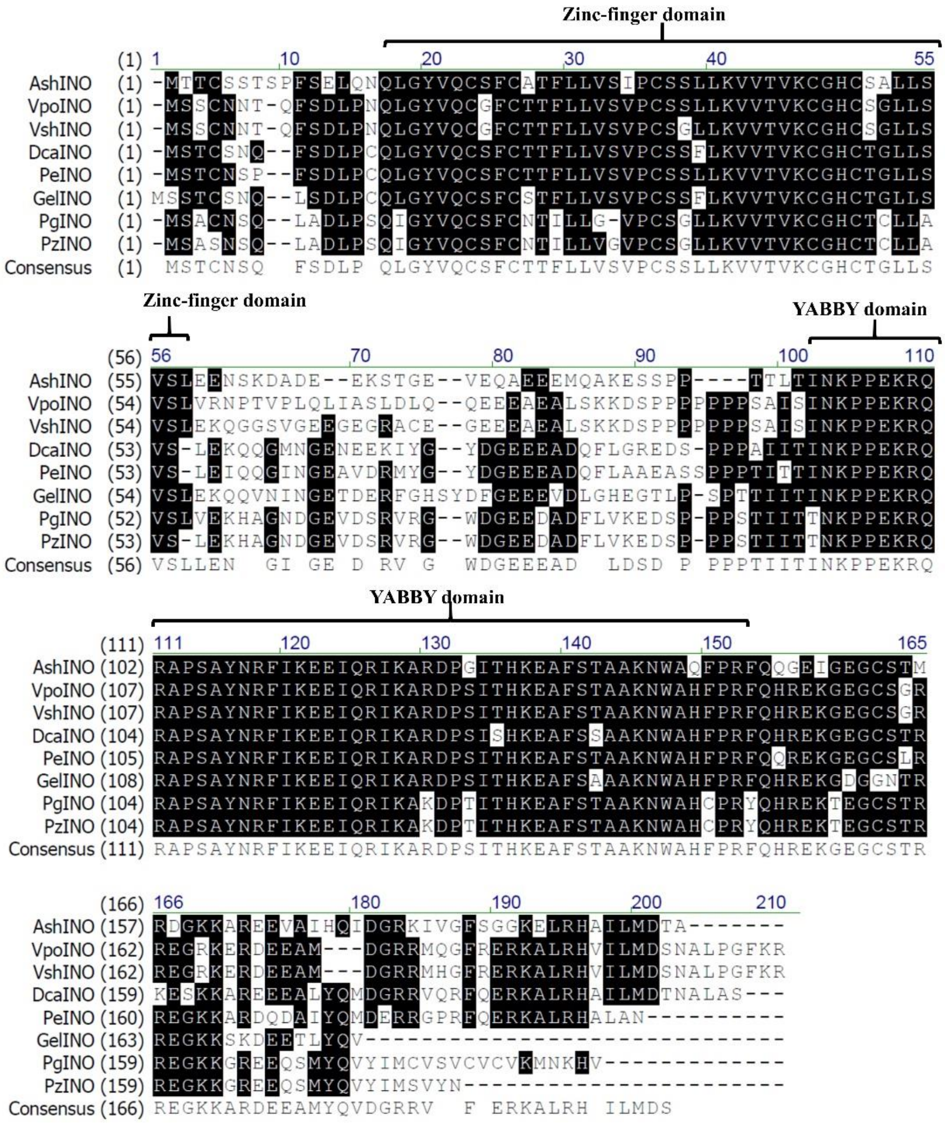

Supplemental Fig. 3. Multiple sequence alignment of INO related protein among eight orchid species.

Multiple sequence alignment of the amino acid sequences of INO proteins was performed by using ClustalW with default settings. The black shade indicates identical amino acids. Two highly conserved C2C2 zinc-finger domain and YABBY domain are marked with black brackets. Gel, *G. elata*; Vpo, *V. planifolia*; Pe, *P. equestris*; Vsh, *V. shenzhenica*; Pg, *P. guangdongensis*; Pz, *P. zijinensis*; Dca, *D. catenatum*; Ash, *A. shenzhenica*. Accession numbers are listed in Table S1.

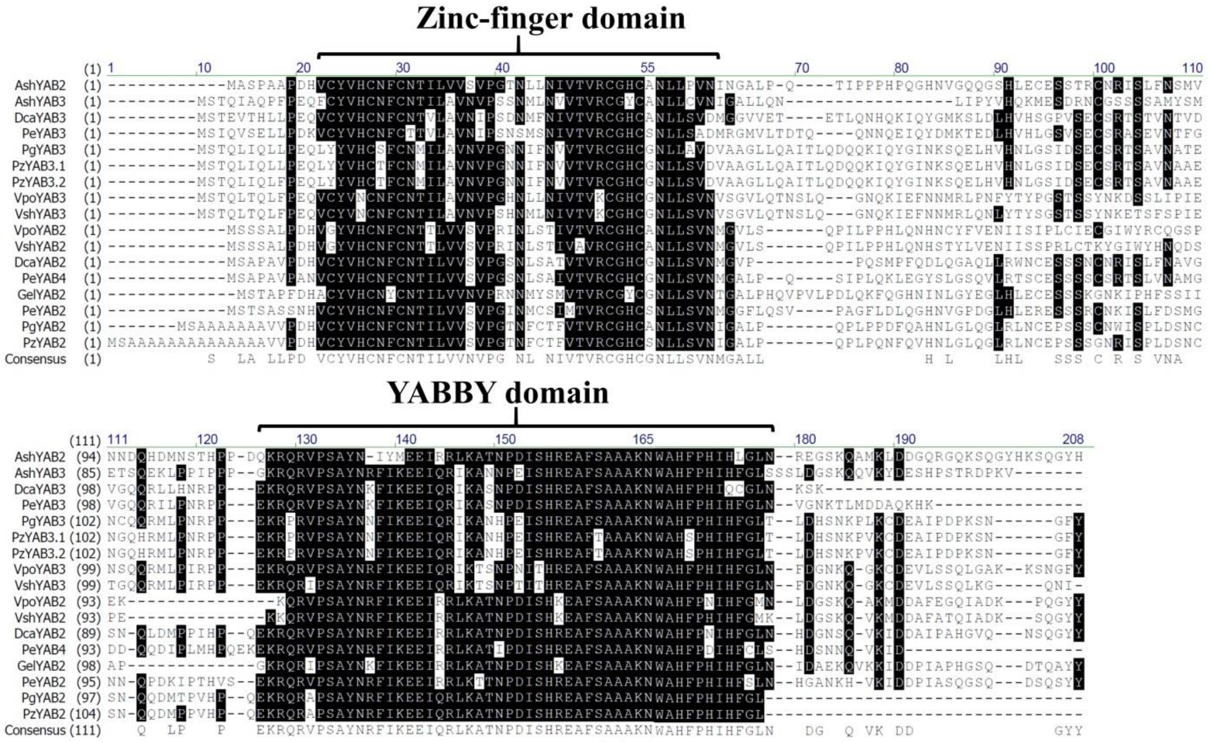

Supplemental Fig. 4. Multiple sequence alignment of YAB2 related protein among eight orchid species.

Multiple sequence alignment of the amino acid sequences of YAB2 proteins was performed by using ClustalW with default settings. The black shade indicates identical amino acids. Two highly conserved C2C2 zinc-finger domain and YABBY domain are marked with black brackets. Gel, *G. elata*; Vpo, *V. planifolia*; Pe, *P. equestris*; Vsh, *V. shenzhenica*; Pg, *P. guangdongensis*; Pz, *P. zijinensis*; Dca, *D. catenatum*; Ash, *A. shenzhenica*. Accession numbers are listed in Table S1.

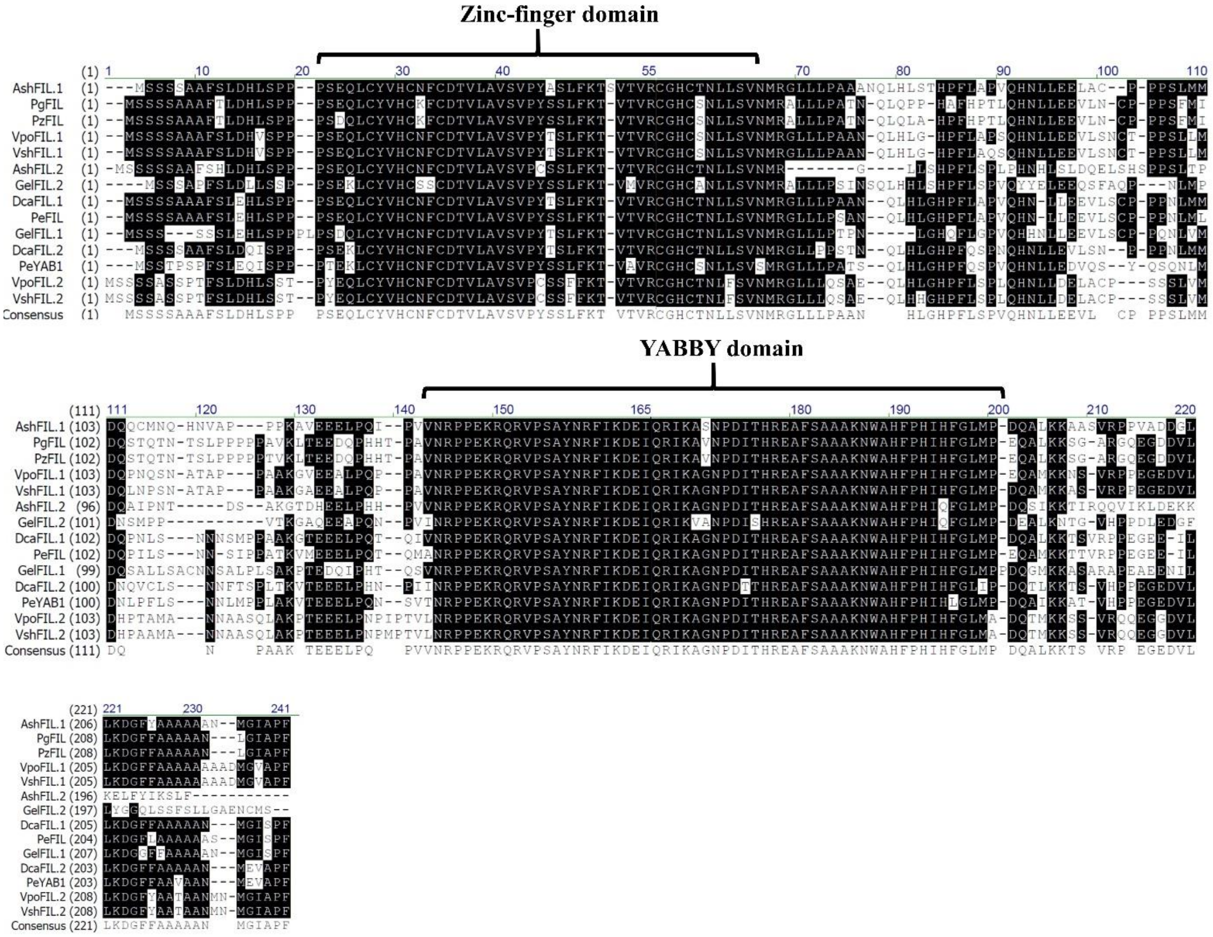

Supplemental Fig. 5. Multiple sequence alignment of FIL related protein among eight orchid species.

Multiple sequence alignment of the amino acid sequences of FIL proteins was performed by using ClustalW with default settings. The black shade indicates identical amino acids. Two highly conserved C2C2 zinc-finger domain and YABBY domain are marked with black brackets. Gel, *G. elata*; Vpo, *V. planifolia*; Pe, *P. equestris*; Vsh, *V. shenzhenica*; Pg, *P. guangdongensis*; Pz, *P. zijinensis*; Dca, *D. catenatum*; Ash, *A. shenzhenica*. Accession numbers are listed in Table S1.

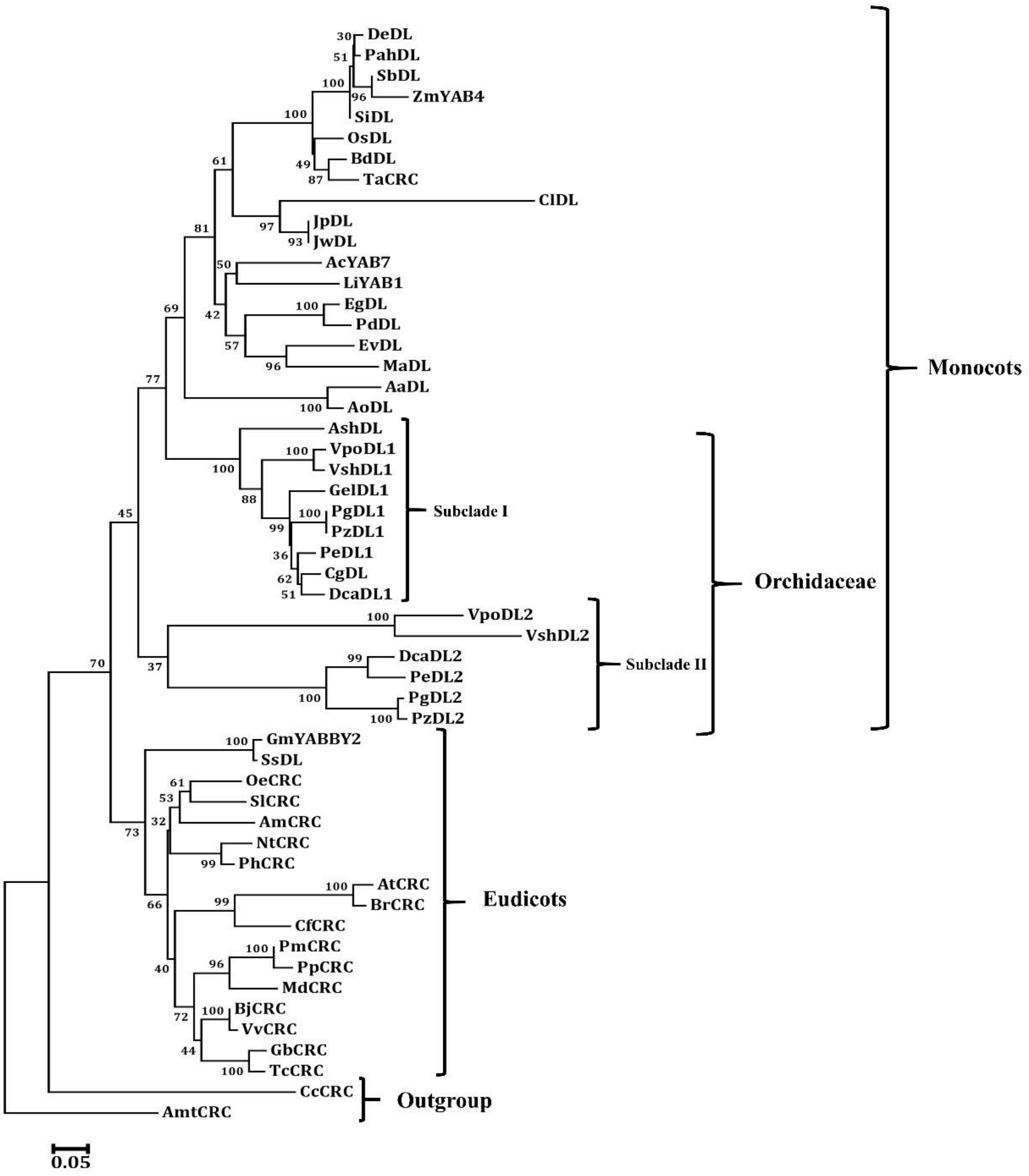

Supplemental Fig. 6. Phylogenetic tree of CRC/DL protein from the angiosperms.

The phylogenetic tree was constructed with neighbor-joining (NJ) method in MEGA 6.0 software. Bootstrap analysis was conducted with 1,000 replications. The protein accession numbers for related proteins are listed in Supplementary Table S1.

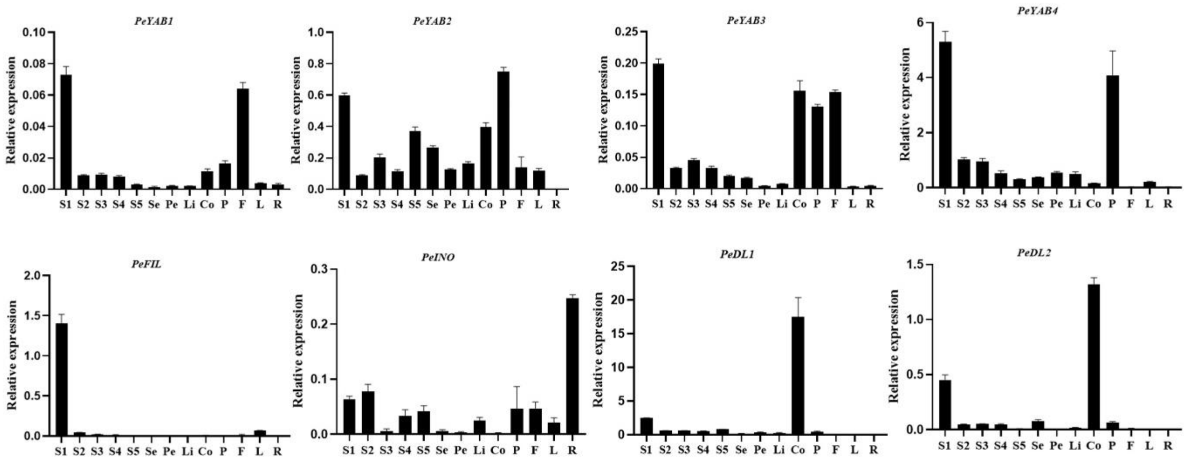

Supplemental Fig. 7. Quantitative real-time PCR analysis of expressions of *PeYABBY* genes in *P. aphrodite* subsp. *formosana* at various organs.

Expression patterns of *PeYABBY* related genes in various plant organs. qRT-PCR data were normalized using *Phalaenopsis PeActin* (PACT4, AY134752) gene. B1-B5, stage 1 to stage 5 floral bud; Se, sepals; Pe, petals; Li, lip; Co, column (gynostemium); P, pedicel; F, floral stalk; L, leaf; R, root. PCR products amplified with primers are listed in Supplementary Table S2.

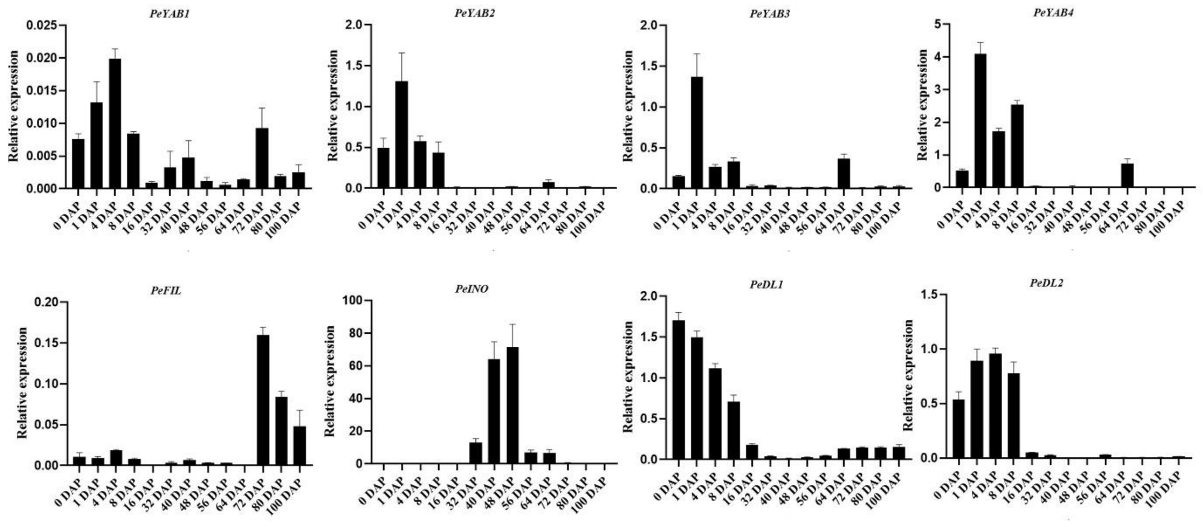

Supplemental Fig. 8. Quantitative real-time PCR analysis of expressions of *PeYABBY* genes in *P. aphrodite* subsp. *formosana* at various ovule developmental stages.

Expression patterns of *PeYABBY* related genes at various ovule developmental stages. qRT-PCR data were normalized using *Phalaenopsis PeActin* (PACT4, AY134752) gene. DAP, days after pollination. PCR products amplified with primers are listed in Supplementary Table S2.

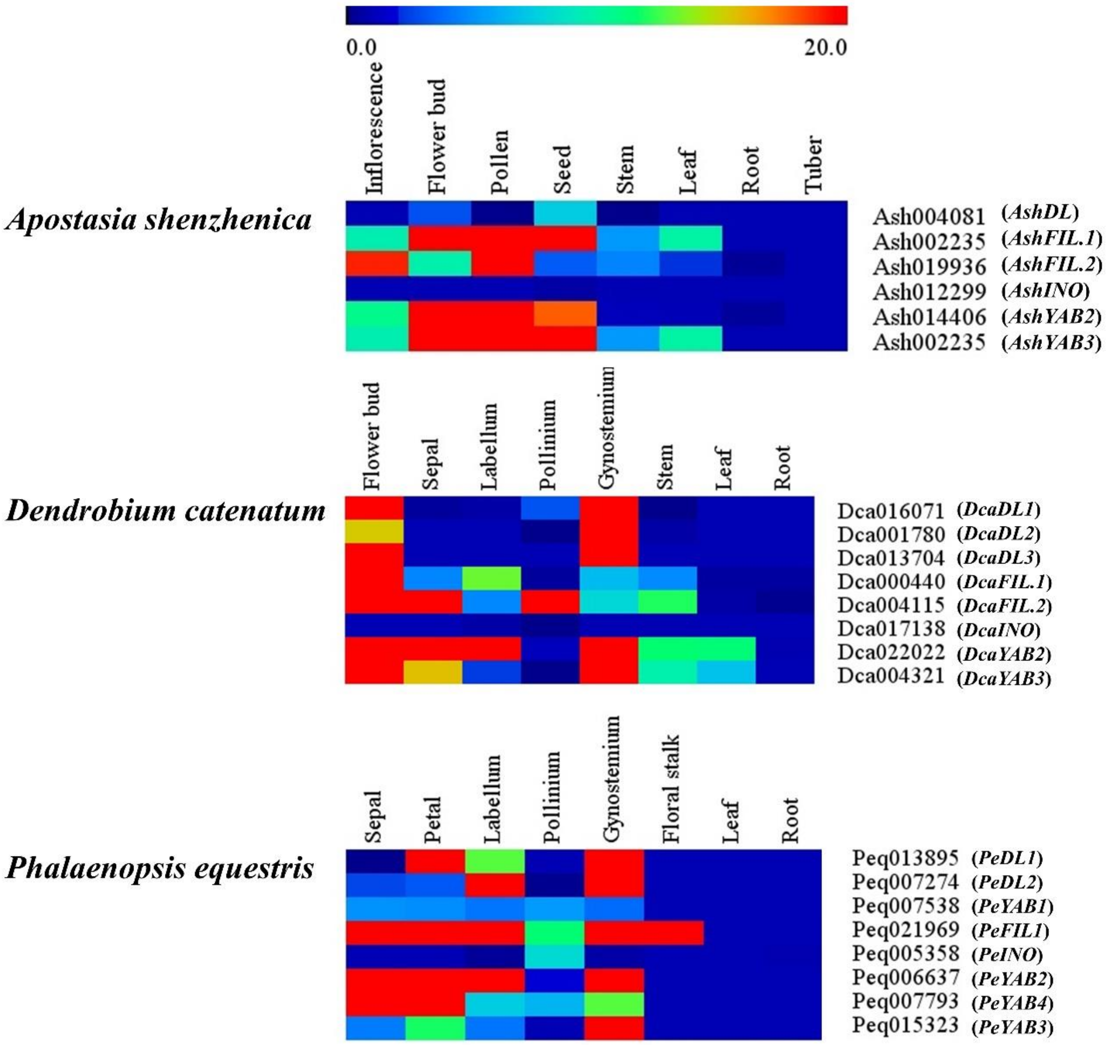

Supplemental Fig. 9. Expression patterns of *YABBY* genes in different tissues of *Apostasia*, *Dendrobium* and *Phalaenopsis*.

The heat map was created based on the FPKM value of orchid YABBY genes. Differential gene expression is shown as per the color scale.
