# Supplementary material for: Genome-Wide Identification of YABBY Genes in Orchidaceae and Their Expression Patterns in Phalaenopsis Orchid
Source: Genes (Basel). 2020 Aug 19;11(9):955. doi: 10.3390/genes11090955 (PMC7563141; doi:10.3390/genes11090955)
Supplement: Supplementary file 1 [file genes-11-00955-s001.zip › genes-872759-suppl/Supplementary_File_3-Table_S2.pdf]

Supplemental Table S2. List of primers used in this work.

Primer Name	Sequence (5'-3')
**Used for real-time RT-PCR**	
PeDL1 qRT F'	5'TCACCAGTTGGGATTTCAGGAT3'
PeDL1 qRT R'	5'ATGGCAAGCGATGCTTCTTC3'
PeDL2 qRT F'	5'TCGGCAGCTACTGAAGACATGA3'
PeDL2 qRT R'	5'ATGTCAGGCTGGGCAGCTT3'
PeINO qRT F'	5'CAGCAAAGAATTGGGCGCAT3'
PeINO qRT R'	5'GGCCCTCTTCTTTCGTCCATC3'
PeYB1 qRT F'	5'CCCATTTTCCCCACATCCA3'
PeYB1 qRT R'	5'GCACGGTGGCCTTCTTTATG3'
PeYB2 qRT F'	5'ATGGCTTACATTTGGAACGAGAA3'
PeYB2 qRT R'	5'GCCCATCGAATCAAAGAGTGA3'
PeYB3 qRT F'	5'GTAGGGCATCAGAAGTCAACACAT3'
PeYB3 qRT R'	5'TGGGCGATTTGGCAGAA3'
PeYAB4 qRT F'	5'TCAATGCCATGGGCGATGAT3'
PeYAB4 qRT R'	5'CCGGAATTGTTGCCTTTAGCC3'
PeFIL qRT F'	5'GACGAAATCCAACGCATCAAA3'
PeFIL qRT R'	5'CGCTGAATGCTTCTCTGTGAGT3'
PeActin4 qRT F'	5'TTGTGAGCAACTGGGATG3'
PeActin4 qRT R'	5'GCC ACG CGAAGTTCATTG3'
